# Genetic variants in m5C modification core genes are associated with the risk of Chinese pediatric acute lymphoblastic leukemia: A five-center case–control study

**DOI:** 10.3389/fonc.2022.1082525

**Published:** 2023-01-09

**Authors:** Xueliang Wang, Decheng Deng, Yaping Yan, Mansi Cai, Xiaodan Liu, Ailing Luo, Shanshan Liu, Xiaohong Zhang, Hua Jiang, Xiaoping Liu

**Affiliations:** ^1^ Department of Hematology/Oncology, Guangzhou Institute of Pediatrics, Guangzhou Women and Children’s Medical Center, Guangzhou Medical University, Guangdong Province Clinical Research Center for Child Health, Guangzhou, Guangdong, China; ^2^ Division of Birth Cohort Study, Guangzhou Women and Children’s Medical Center, Guangzhou Medical University, Guangdong Province Clinical Research Center for Child Health, Guangzhou, Guangdong, China

**Keywords:** ALL, pediatric, 5-methylcytosine, susceptibility, polymorphism

## Abstract

**Objective:**

To explore the functions of the polymorphisms in 5-methylcytosine (m5C) modification-related coding genes on the susceptibility of pediatric acute lymphoblastic leukemia (ALL).

**Methods:**

Case–control study and multinomial logistic regression analysis were performed to construct models to evaluate the susceptibility of pediatric ALL. The relationship between five functional SNPs in m5C modification-coding genes and pediatric ALL risk was analyzed. Genotyping of 808 cases and 1,340 healthy samples from South China was identified using a TaqMan assay; odds ratios (ORs) and 95% confidence intervals (CIs) were calculated to estimate the relationship between the five selected SNPs and pediatric ALL susceptibility.

**Results:**

Among the five analyzed SNPs, *NOL1* rs3764909 and *NSUN4* rs10252 variants significantly increased the susceptibility of pediatric ALL, while *NSUN3* rs7653521, *NSUN5* rs1880948, and *NSUN6* rs3740102 variants were not associated with the risk of ALL. Stratification analyses demonstrated that *NOL1* rs3764909 C>A exhibited a significant association with increased pediatric ALL risk in subgroups of common B ALL, pre-B ALL, T-cell ALL, low and middle risk, other gene fusion types, non-gene fusion, hypodiploid, normal diploid, primitive lymphocytes in marrow < 5% on week 12, and minimal residual disease (MRD) <0.01% on week 12 after induced therapy; *NSUN4* rs10252 G>A was related to increased risk of ALL children in subgroups of age ≥ 120 months, normal white blood cell (WBC) number, middle risk, non-gene fusion, MRD ≥ 0.01 on days 15–19, and primitive lymphocytes in marrow < 5% on day 33 after induced therapy. Compared with the reference haplotype CAGTA, children who harbored haplotypes CCGTG and ACATA were remarkably related to increased ALL susceptibility. rs3764909 and rs10252 varieties of alleles were not associated with MRD levels after the selected chemotherapeutics.

**Conclusions:**

In conclusion, *NOL1* rs3764909 and *NSUN4* rs10252 variants were enhanced by pediatric ALL risk and were suggested to be potential biomarkers for pediatric ALL.

## Introduction

Acute lymphoblastic leukemia (ALL) is the most common malignant tumor in children and adolescents (1–3). Despite the heterogeneity, the clinical cure rate of childhood ALL is over 85%. Combination chemotherapy and allogeneic hematopoietic cell transplantation is the main treatment of ALL. In order to obtain the best treatment effect, different chemotherapy regimens are formulated according to the risk of recurrence. Even though these efforts were made, there are still a significant proportion of children with ALL having a high risk of relapse (4). Several genetic factors are verified to enhance the risk of pediatric ALL, but a certain percentage of children were not recognized to inherit risk genetic factors (5). Plenty of studies have discovered polymorphic variants in genes that are connected with an elevated risk of ALL (6–8).

In recent years, abundant studies have revealed that epigenetic regulation participates in the initiation and procession of tumors. The role and regulatory mechanism of RNA methylation in tumors have attracted the close attention of researchers. RNA methylation refers to the chemical modification of RNA methyladenine by the selective addition of methyl groups under the catalysis of methyltransferase. Common RNA methylations include several sites (9). A number of m6A-methylated genes take part in the carcinogenesis of leukemia. m6A methyltransferase *METTL14* was demonstrated to promote leukemogenesis *via* mRNA m6A modification (10). m6A demethylase FTO attenuates aerobic glycolysis and accelerates leukemia. Our previous studies identified that genetic variants in m6A methyltransferase *METTL3* and *METTL14* were associated with the increased risk of pediatric ALL (11, 12).

As we know, *N* ^6^-methyladenosine is the most common modification of RNA methylation, and 5-methylcytosine (m5C) is another common and conserved modification in RNA, including mRNAs and non-coding RNAs. m5C regulates RNA stability assembly and translation as well as m6A (13). The enzymes modulating m5C of RNAs can be functionally classified into writers, erasers, and readers. Methyltransferases (writers) can install m5C on RNA. The reported m5C writers include NSUN1–7 and DNMT2 (14). Ten-eleven translocation family proteins (TETs) can oxidize 5-methylcytosine to cytosine-5-hydroxymethylation (hm5C), so these are regarded as “erasers” for m5C (15). As for “reader”, only YBX1 and ALYREF have been identified as recognition proteins for m5C modification sites at present (16). NOL1/NOP2/SUN family is also documented as m5C methyltransferase to regulate RNA stability and functions (14). These RNA modifiers can regulate the expression of various oncogenes and promote tumorigenesis and development. In addition, methyltransferases are abnormally expressed in a variety of tumors and have been used to predict the prognosis of patients. There is only one available study on the epidemiological assessment of single-nucleotide polymorphisms (SNPs) in the m5C modification core gene. Chen and Cao et al. performed a case–control study and verified that m5C modification genes were related to survival and chemotherapy efficacy of colorectal cancer. Two SNPs of *YBX1* gene, rs10890208 and rs3862218, may predict a reduction by using the Cox regression model to analyze the association between 13 candidate SNPs of m5C modifier gene and overall survival (OS) of colorectal cancer (CRC) after chemotherapy (17). However, the role of SNPs in the m5C methyltransferase gene in ALL risk has not been reported. Because of the evidence that cells regulated by the m5C methyltransferase gene promote tumorigenesis, we conducted a case–control study to explore the association of genetic variations in m5C modification-coding genes with the risk to pediatric ALL in China.

## Materials and methods

### Study subjects

A total of 808 pediatric ALL cases and 1,340 age-matched, gender-matched, and ethnicity-matched control samples from South China were enrolled from January 2017 to May 2019 in this study, as summarized in our previous studies (11, 12). All children were diagnosed with ALL by at least two hematologists. The control samples were free from hematological diseases, malignancy, or any type of autoimmune disorder.

### SNP selection

The potentially functional SNPs in five m5C methyltransferases were selected as previously described, and the protocol was as follows; the National Center for Biotechnology Information (NCBI) dbSNP database and SNP info (https://snpinfo.niehs.nih.gov/) were used. The selected SNPs should fulfill the following criteria: (1) the minor allele frequency (MAF) was >5% of Chinese Han subjects in HapMap and (2) located in the exon, 5′ untranslated regions (5′ UTR), and 3′ UTR of genes, which were predicted to be potential functional; (3) each SNP should be in low linkage disequilibrium (R^2^ < 0.8). Five SNPs were selected (*NOL1* rs3764909, *NSUN3* rs7653521, *NSUN4* rs10252, *NSUN5* rs1880948, and *NSUN6* rs3740102). rs3764909 is located in the exonic region of *NOL1* and might be a transcriptional factor binding site. rs7653521 is located in the exonic region of *NSUN3* and was predicted to have the potential to bind transcriptional factors. rs10252 is located in the exon of *NSUN4* and was predicted as a miRNA binding site. rs1880948 is located upstream of *NSUN5* transcriptional start site and may be a transcriptional factor binding site. rs3740102 is located in an exon of *NSUN6* and was predicted to be a transcriptional factor binding site.

### SNP genotyping

Peripheral blood genomic DNA was extracted using the QIAamp DNA blood mini kit (QIAGEN, Valencia, CA, USA). For genotyping, assay probes were purchased from Thermo Fisher (Waltham, MA, USA; TaqMan SNP Assays, 4351379). The detailed information on these assays is presented in **Table S1**. The genotype was identified by TaqMan PCR on an ABI 7900 (Applied Biosystems, Foster City, CA, USA). The conditions of reactions were described previously (11). To ensure the accuracy of these genotyping results, 10% of the samples were randomly selected to be genotyped by a DNA sequencing method. A concordance rate of 100% for the quality control samples was obtained (11).

### Statistical analysis

The compliance of genotypes with the Hardy–Weinberg equilibrium (HWE) in the control group and differences in clinical characteristics between ALL children and healthy children were evaluated using the χ^2^ test. The age- and gender-adjusted odds ratios (ORs) and 95% confidence intervals (CIs) for the association between the SNPs and ALL susceptibility were calculated by multivariate logistic regression analysis. All these analyses were performed using the software SAS v10.0 (SAS Institute, Cary, NC, USA).

## Results

### The association of m5C modification core genes and pediatric ALL risk

Five m5C modification core gene SNPs (*NOL1* rs3764909 C>A, *NSUN3* rs7653521 T>C, *NSUN4* rs10252 G>A, *NSUN5* rs1880948 G>A, and *NSUN6* rs3740102 C>A) were genotyped in 808 pediatric ALL samples and 1,340 age- and gender-matched healthy controls. The five SNPs comply with the HWE in control populations. Single-locus analysis was used to analyze the relationship between the five SNPs and pediatric ALL risk. The *NOL1* rs3764909 (AC/CC versus AA: adjusted OR = 1.684, 95% CI = 1.408–2.014, p < 0.001) and *NSUN4* rs10252 (adjusted OR = 1.140, 95% CI = 1.001–1.298, p = 0.049) variant alleles were associated with an increased risk of ALL. However, there was no association between the remaining polymorphisms, *NSUN3* rs7653521 (OR = 1.067, 95% CI = 0.946–1.203, p = 0.291), *NSUN5* rs1880948 (OR = 0.980, 95% CI = 0.862–1.114, p = 0.759), *NSUN6* rs3740102 (OR = 0.959, 95% CI = 0.825–1.114, p = 0.581), and pediatric ALL risk (**Table 1**).

**Table 1:** Logistic regression analysis of associations between m5C modification key gene polymorphisms and ALL susceptibility.

Genotype	Cases	Controls	p^a^	Crude OR	p	Adjusted OR	p^b^
	(N = 808)	(N = 1,340)		(95% CI)		(95% CI)^b^	
rs3740102 (HWE = 0.0978)							
CC	462 (57.39)	757 (56.66)		1		1	
CA	309 (38.39)	512 (38.32)		0.988 (0.823–1.185)	0.894	0.988 (0.824–1.186)	0.900
AA	34 (4.22)	67 (5.01)		0.830 (0.541–1.275)	0.396	0.836 (0.546–1.287)	0.420
Additive			0.700	0.957 (0.824–1.111)	0.562	0.959 (0.825–1.114)	0.581
Dominant	343 (42.61)	579 (43.34)	0.741	0.971 (0.813–1.158)	0.741	1.146 (0.815–1.160)	0.755
Recessive	771 (95.78)	1,269 (94.99)	0.403	0.835 (0.548–1.274)	0.403	0.842 (0.552–1.286)	0.427
rs3764909 (HWE = 0.2333)							
AA	297 (36.80)	661 (49.55)		1		1	
AC	414 (51.30)	543 (40.70)		1.706 (1.415–2.058)	<0.001	1.701 (1.411–2.052)	<0.001
CC	96 (11.90)	130 (9.75)		1.653 (1.228–2.224)	0.001	1.660 (1.233–2.234)	0.001
Additive			<0.001	0.405 (1.231–1.604)	<0.001	1.406 (1.231–1.805)	<0.001
Dominant	510 (63.20)	673 (50.45)	<0.001	1.687 (1.419–2.017)	<0.001	1.684 (1.408–2.014)	<0.001
Recessive	711 (88.10)	1,204 (90.25)	0.117	1.251 (0.946–1.654)	0.117	1.258 (0.951–1.664)	0.108
rs3776448 (HWE < 0.001)							
TT	501 (62.08)	688 (51.42)		1		1	
TC	23 (2.85)	60 (4.46)		0.527 (0.321–0.864)	0.011	0.523 (0.319–0.858)	0.010
CC	283 (35.07)	590 (44.10)		0.659 (0.549–0.791)	<0.001	0.658 (0.519–790)	<0.001
Additive			<0.001	0.809 (0.738–0.886)	<0.001	0.808 (0.738–0.886)	<0.001
Dominant	524 (35.07)	748 (55.10)	<0.001	1.460 (1.219–1.749)	<0.001	1.462 (1.220–1.751)	<0.001
Recessive	306 (37.92)	650 (48.58)	<0.001	1.547 (1.294–1.848)	<0.001	1.550 (1.297–1.852)	<0.001
rs1880948 (HWE = 0.9796)							
GG	335 (41.61)	540 (40.33)		1		1	
GA	361 (44.84)	621 (46.38)		0.960 (0.771–1.123)	0.752	0.931 (0.772–1.124)	0.458
AA	109 (13.54)	178 (13.29)		0.980 (0.745–1.289)	0.885	0.985 (0.749–1.296)	0.915
Additive			0.557	0.978 (0.860–1.112)	0.733	0.980 (0.862–1.114)	0.759
Dominant	470 (58.39)	799 (59.67)	0.100	0.948 (0.794–1.132)	0.557	0.950 (0.795–1.135)	0.573
Recessive	696 (86.46)	1,161 (86.71)	0.871	1.022 (0.791–1.320)	0.871	1.026 (0.794–1.326)	0.283
rs7653521 (HWE = 0.1783)							
TT	237 (31.91)	392 (29.41)		1		1	
TC	361 (44.68)	639 (47.94)		0.951 (0.774–1.169)	0.634	0.950 (0.773–1.167)	0.625
CC	210 (25.99)	302 (22.66)		1.171 (0.922–1.486)	0.195	1.172 (0.923–1.488)	0.193
Additive			0.175	1.066 (0.946–1.202)	0.294	1.067 (0.946–1.203)	0.291
Dominant	571 (70.67)	941 (70.59)	0.970	1.004 (0.828–1.216)	0.970	1.003 (0.828–1.215)	0.976
Recessive	598 (74.01)	1,031 (77.34)	0.080	1.199 (0.979–1.469)	0.080	1.201 (0.980–1.471)	0.077
rs10252 (HWE = 0.3448)							
AA	342 (42.64)	628 (46.90)		1		1	
AG	361 (45.01)	567 (42.35)		1.151 (0.956–1.386)	0.139	1.151 (0.956–1.387)	0.137
GG	99 (12.34)	144 (10.75)		1.243 (0.932–1.656)	0.139	1.247 (0.936–1.663)	0.132
Additive			0.139	1.138 (0.999–1.295)	0.051	1.140 (1.001–1.298)	0.049
Dominant	460 (57.36)	711 (53.10)	0.055	1.188 (0.996–1.417)	0.056	1.190 (0.997–1.420)	0.054
Recessive	703 (87.66)	1,195 (89.25)	0.262	1.169 (0.890–1.534)	0.262	1.173 (0.893–1.541)	0.251

ALL, acute lymphoblastic leukemia; HWE, Hardy–Weinberg equilibrium.

a χ^2^ test for genotype distributions between leukemia cases and cancer-free controls.

b Adjusted for age and gender.

### Stratification analysis of rs3764909 and rs10252 with ALL susceptibility

SNPs rs3764909 C>A and rs10252 T>C with statistically significant differences were stratified according to age, gender, white blood cell (WBC), immunophenotype, gene infusion, karyotype, primitive lymphocytes in the marrow, minimal residual disease (MRD), and relapse (**Table 2**). *NOL1* rs3764909 AC/CC increased ALL risk in children aged <120 months (adjusted OR = 1.592, 95% CI = 1.319–1.923, p < 0.001), children aged ≥120 months (adjusted OR = 2.759, 95% CI = 1.568–4.855, p < 0.001), female (adjusted OR = 1.785, 95% CI = 1.408–2.265, p < 0.001), male (adjusted OR = 1.860, 95% CI = 1.381–2.505, p < 0.001), number of WBC > 10 × 10^7^ (adjusted OR = 1.811, 95% CI = 1.417–2.315, p < 0.001), normal WBC number (adjusted OR = 1.668, 95% CI = 1.322–2.106, p < 0.001), common B ALL (adjusted OR = 3.323, 95% CI = 2.495–4.426, p < 0.001), pre-B ALL (adjusted OR = 1.566, 95% CI = 1.125–2.181, p = 0.008), T-ALL (adjusted OR = 1.890 95% CI = 1.132–3.155, p = 0.015), low-risk ALL (adjusted OR = 1.705, 95% CI = 1.278–2.276, p < 0.001), middle-risk ALL (adjusted OR = 2.074, 95% CI = 1.630–2.640, p < 0.001), other gene fusion types (adjusted OR = 4.806, 95% CI = 1.623–14.242, p = 0.005), non-gene fusion (adjusted OR = 4.524, 95% CI = 2.896–7.065, p < 0.001), hypodiploid (adjusted OR = 3.330, 95% CI = 1.327–8.539, p = 0.010), normal diploid (OR = 3.403, 95% CI = 2.364–4.898, p < 0.001), primitive/naïve lymphocytes in marrow ≥ 5% on days 15–19 (adjusted OR = 1.718, 95% CI = 1.395–2.116, p < 0.001) and day 33 (adjusted OR = 1.466, 95% CI = 1.185–1.813, p < 0.001), and <5% (adjusted OR = 1.623, 95% CI = 1.247–2.112, p < 0.001) on days 15–19, day 33 (adjusted OR = 2.082, 95% CI = 1.603–2.688, p < 0.001), and week 12 (adjusted OR = 2.704, 95% CI = 2.153–3.397, p < 0.001) after induced therapy with MRD ≥ 0.01% on days 15–19 (adjusted OR = 1.952, 95% CI = 1.537–2.479, p < 0.001) and day 33 (adjusted OR = 1.729, 95% CI = 1.144–2.613, p = 0.009), with MRD < 0.01% on days 15–19 (adjusted OR = 1.486, 95% CI = 1.190–1.854, p < 0.001), day 33 (adjusted OR = 1.677, 95% CI = 1.391–2.023, p < 0.001), and week 12 (adjusted OR = 1.710, 95% CI = 1.428–3.397, p < 0.001) after induced chemotherapy, with relapse (adjusted OR = 2.332, 95% CI = 1.058–5.138, p = 0.036) and no relapse (adjusted OR = 1.262, 95% CI = 1.034–1.541, p = 0.022).

**Table 2:** Stratification analysis of m5C-related gene polymorphisms with ALL susceptibility.

Variables	rs3764909		Adjusted OR^a^	p^a^	rs10252		Adjusted OR^a^	p^a^
	(cases/controls)				(cases/controls)			
	AA	AC/CC	(95% CI)		AA	AG/GG	(95% CI)	
Age, month								
<120	270/661	446/673	1.592 (1.319–1.923)	<0.001	311/628	400/711	1.113 (0.924–1.341)	0.259
≥120	27/661	64/673	2.759 (1.568–4.855)	0.000	31/628	60/711	2.056 (1.184–3.570)	0.010
Gender								
Female	156/661	291/673	1.785 (1.408–2.265)	<0.001	183/628	260/711	1.205 (0.954–1.521)	0.118
Male	108/661	200/673	1.860 (1.381–2.505)	<0.001	135/628	172/711	1.252 (0.936–1.674)	0.129
WBC								
>10	122/661	225/673	1.811 (1.417–2.315)	<0.001	156/628	188/711	1.065 (0.839–1.352)	0.603
≤10	143/661	244/673	1.668 (1.322–2.106)	<0.001	158/628	228/711	1.286 (1.022–1.619)	0.032
Immunophenotyping								
Pro B	119/661	108/673	0.885 (0.668–1.175)	0.399	107/628	119/711	0.983 (0.740–1.305)	0.905
Common B	71/661	238/673	3.323 (2.495–4.426)	<0.001	134/628	173/711	1.137 (0.885–1.460)	0.316
Pre-B	64/661	102/673	1.566 (1.125–2.181)	0.008	67/628	98/711	1.304 (0.938–1.813)	0.114
T ALL	24/661	45/673	1.890 (1.132–3.155)	0.015	26/628	42/711	1.424 (0.858–2.364)	0.172
Mix	3/661	2/673	0.676 (0.112–4.082)	0.670	2/628	3/711	1.369 (0.226–8.300)	0.733
Risk								
Low	85/661	150/673	1.705 (1.278–2.276)	<0.001	98/628	136/711	1.241 (0.915–1.612)	0.174
Normalized	18/661	13/673	0.689 (0.334–1.419)	0.312	15/628	15/711	0.878 (0.425–1.814)	0.726
Middle	123/661	258/673	2.074 (1.630–2.640)	<0.001	156/628	224/711	1.285 (1.019–1.621)	0.034
High	35/661	54/673	1.525 (0.983–2.368)	0.060	44/628	43/711	0.861 (0.557–1.331)	0.501
Gene fusion type								
BCR-ABL	10/661	18/673	1.798 (0.820–3.942)	0.143	14/628	14/711	0.854 (0.401–1.815)	0.681
E2A-PBX	11/661	13/673	1.139 (0.506–2.565)	0.753	9/628	15/711	1.486 (0.645–1.424)	0.353
MLL	5/661	11/673	2.187 (0.754–0.339)	0.150	6/628	10/711	1.427 (0.514–3.962)	0.495
SIL-TAL	1/661	7/673	7.003 (0.857–57.188)	0.069	1/628	7/711	0.964 (0.729–46.782)	0.096
TCF3-PBX1	0/661	9/673	>999.999 (<0.001, >999.999)	0.938	3/628	6/711	1.964 (0.485–7.957)	0.344
TEL/ETV6	0/661	7/673	>999.999 (<0.001, >999.999)	0.952	3/628	4/711	1.153 (0.257–5.176)	0.853
TEL-AML	41/661	47/673	1.119 (0.726–1.725)	0.610	36/628	53/711	1.122 (0.851–2.055)	0.214
Others	4/661	19/673	4.806 (1.623–14.242)	0.005	11/628	12/711	0.989 (0.432–2.264)	0.978
Non	24/661	115/673	4.524 (2.896–7.065)	<0.001	48/628	92/711	1.684 (1.169–2.428)	0.005
Karyotype								
Hypo-diploid	6/661	20/673	3.330 (1.327–8.539)	0.010	11/628	15/711	1.236 (0.562–2.721)	0.598
Normal diploid	41/661	142/673	3.403 (2.364–4.898)	<0.001	81/628	102/711	1.129 (0.827–1.542)	0.445
Abnormal diploid	100/661	134/673	1.314 (0.993–1.740)	0.056	101/628	133/711	1.168 (0.882–1.546)	0.278
Low hyperdiploid	10/661	19/673	1.896 (0.874–4.116)	0.106	9/628	20/711	2.031 (0.916–4.503)	0.081
High hyperdiploid	31/661	34/673	0.905 (0.561–1.460)	0.683	27/628	43/711	1.429 (0.872–2.343)	0.157
Primitive/naïve lymphocytes in marrow (%, 15–19 days)								
<5	107/661	176/673	1.623 (1.247–2.112)	<0.001	115/628	166/711	1.273 (0.980–1.653)	0.971
≥5	190/661	334/673	1.718 (1.395–2.116)	<0.001	227/628	294/711	1.152 (0.939–1.413)	0.174
MRD in marrow (%, 15–19 days)								
<0.01	170/661	257/673	1.486 (1.190–1.854)	<0.001	186/628	239/711	1.133 (0.909–1.411)	0.267
≥0.01	127/661	253/673	1.952 (1.537–2.479)	<0.001	156/628	221/711	1.264 (1.002–1.594)	0.048
Primitive/naïve lymphocytes in marrow (%, 33 days)								
<5	106/661	224/673	2.082 (1.603–2.688)	<0.001	134/628	194/711	1.281 (1.002–1.837)	0.048
≥5	191/661	286/673	1.466 (1.185–1.813)	0.000	208/628	266/711	1.136 (0.920–1.403)	0.237
MRD in marrow (%, 33 days)								
<0.01	259/661	443/673	1.677 (1.1.391–2.023)	<0.001	288/628	409/711	1.256 (1.044–1.512)	0.016
≥0.01	38/661	67/673	1.729 (1.144–2.613)	0.009	54/628	51/711	0.841 (0.565–1.253)	0.395
Primitive/naïve lymphocytes in marrow (%, 12 weeks)								
<5	130/661	356/673	2.704 (2.153–3.397)	<0.001	205/628	279/711	1.200 (0.972–1.381)	0.089
≥5	167/661	154/673	0.900 (0.705–1.150)	0.400	137/628	181/711	1.173 (0.916–1.501)	0.206
MRD in marrow (%, 12 weeks)								
<0.01	289/661	504/673	1.710 (1.428–2.048)	<0.001	406/628	381/711	1.190 (0.996–1.421)	0.055
≥0.01	8/661	6/673	0.761 (0.262–2.213)	0.616	6/628	8/711	1.239 (0.426–3.606)	0.695
Relapse								
−	243/661	314/673	1.262 (1.034–1.541)	0.022	236/628	317/711	1.204 (0.985–1.472)	0.069
+	9/661	21/673	2.332 (1.058–5.138)	0.036	18/628	12/711	0.571 (0.273–1.198)	0.139

ALL, acute lymphoblastic leukemia; WBC, white blood cell; MRD, minimal residual disease.

a Adjusted for age and gender.

-: no ALL relapse.

+: ALL relapse.

*NSUN4* rs10252 AG/GG also increased ALL risk in children of age ≥ 120 months (adjusted OR = 2.056, 95% CI = 1.184–3.570, p = 0.010), normal WBC number (adjusted OR = 1.286, 95% CI = 1.022–1.619, p = 0.032), middle risk (adjusted OR = 1.285, 95% CI = 1.019–1.621, p = 0.034), non-gene fusion (adjusted OR = 1.684, 95% CI = 1.169–2.428, p = 0.005), primitive/naïve lymphocyte <5% on days 15–19 (adjusted OR = 1.281, 95% CI = 1.002–1.837, p = 0.048), MRD ≥ 0.01% on days 15–19 (adjusted OR = 1.264 95% CI = 1.002–1.594 p = 0.048), and MRD < 0.01% (adjusted OR = 1.256, 95% CI = 1.044–1.512, p = 0.016) after induced chemotherapy.

### Haplotype analysis of SNPs in m5C methyltransferase coding gene correlated with pediatric ALL susceptibility

Furtherly, whether the haplotypes of *NOL1* rs3764909, *NSUN3* rs7653521, *NSUN4* rs10252, *NSUN5* rs1880948, and *NSUN6* rs3740102 are linked to pediatric ALL susceptibility were evaluated. The wild-type allele CAGTA was considered as the reference group The results showed that children with haplotypes CCGTG (adjusted OR = 2.035, 95% CI = 1.095–3.782, p = 0.025) and ACATA (adjusted OR = 3.169, 95% CI = 1.455–6.899, p = 0.004) would have enhanced ALL susceptibility (**Table 3**).

**Table 3:** Association between inferred haplotypes of the m5C-related genes and pediatric ALL risk.

Haplotypes	Cases (n = 1,594)	Controls (n = 2,644)	Crude OR (95% CI)	p	Adjusted OR (95% CI)	p
	No.%	No.%				
CAGTA	386 (24.22)	708 (26.78)	1.000		1.000	
ACGTG	14 (0.88)	18 (0.68)	0.984 (0.662–1.463)	0.936	0.988 (0.664–1.469)	0.951
CAGTG	53 (3.95)	106 (4.01)	1.031 (0.650–1.732)	0.812	1.065 (0.653–1.738)	0.801
CAATG	20 (1.25)	42 (1.59)	0.850 (0.443–1.634)	0.627	0.861 (0.448–1.655)	0.653
AAGTG	10 (0.63)	28 (1.06)	0.638 (0.282–1.441)	0.280	0.641 (0.284–1.447)	0.284
AAATG	15 (0.94)	32 (1.21)	0.837 (0.407–1.720)	0.628	0.844 (0.411–1.735)	0.644
ACATG	13 (0.82)	26 (0.98)	0.893 (0.415–1.920)	0.772	0.897 (0.417–1.929)	0.781
CCATG	29 (1.82)	34 (1.29)	1.523 (0.817–2.840)	0.186	1.531 (0.821–2.855)	0.180
CCGTA	68 (4.27)	91 (3.44)	1.334 (0.816–2.181)	0.250	1.342 (0.821–2.194)	0.241
ACGTA	10 (0.63)	25 (0.95)	0.714 (0.313–1.630)	0.424	0.715 (0.313–1.631)	0.425
CCGTG	34 (2.13)	30 (1.13)	2.024 (1.089–3.760)	0.026	2.035 (1.095–3.782)	0.025
CAATA	75 (4.71)	135 (5.11)	0.992 (0.619–1.590)	0.974	0.999 (0.623–1.601)	0.997
AAGTA	19 (1.19)	50 (1.89)	0.679 (0.355–1.299)	0.242	0.683 (0.357–1.308)	0.260
AAATA	18 (1.13)	25 (0.95)	1.286 (0.630–2.626)	0.490	1.282 (0.628–2.618)	0.495
ACATA	23 (1.44)	13 (0.49)	3.158 (1.451–6.876)	0.004	3.169 (1.455–6.899)	0.004
CCATA	23 (1.44)	47 (1.78)	0.874 (0.467–1.634)	0.673	0.874 (0.468–1.634)	0.674
CCGCG	53 (3.32)	60 (2.27)	1.577 (0.930–2.676)	0.091	0.594 (0.940–2.706)	0.084
ACGCG	35 (2.20)	47 (1.78)	1.330 (0.746–2.370)	0.334	1.333 (0.748–2.377)	0.330
CAGCG	45 (2.82)	60 (2.27)	1.339 (0.780–2.299)	0.289	1.350 (0.786–2.319)	0.276
CAACG	44 (2.76)	87 (3.29)	0.903 (0.536–1.525)	0.703	0.912 (0.540–1.540)	0.731
AAGCG	23 (1.44)	47 (1.78)	0.874 (0.467–1.634)	0.673	0.878 (0.469–1.641)	0.682
AAACG	30 (1.88)	58 (2.19)	0.924 (0.517–1.650)	0.789	0.930 (0.520–1.662)	0.807
ACACG	56 (3.51)	69 (2.61)	1.449 (0.864–2.430)	0.159	1.461 (0.872–2.451)	0.150
CCACG	70 (4.39)	96 (3.63)	1.302 (0.800–2.120)	0.289	1.302 (0.800–2.120)	0.289
CCGCA	60 (3.76)	80 (3.03)	1.339 (0.809–2.219)	0.257	1.343 (0.811–2.225)	0.252
ACGCA	32 (2.01)	33 (1.25)	1.732 (0.936–3.205)	0.081	1.735 (0.937–3.211)	0.080
CAGCA	151 (9.47)	244 (9.23)	1.105 (0.720–1.697)	0.648	1.111 (0.723–1.706)	0.631
CAACA	54 (3.39)	112 (4.24)	0.861 (0.523–1.417)	0.556	0.864 (0.525–1.421)	0.564
AAGCA	18 (1.13)	54 (2.04)	0.596 (0.310–1.145)	0.120	0.600 (0.312–1.154)	0.126
AAACA	24 (1.51)	60 (2.27)	0.714 (0.390–1.309)	0.276	0.720 (0.393–1.320)	0.289
ACACA	37 (2.32)	52 (1.97)	1.271 (0.721–2.238)	0.407	1.278 (0.726–2.251)	0.396
CCACA	42 (2.63)	75 (2.84)	0.928 (0.639–1.360)	0.701	0.922 (0.629–1.353)	0.679

### The influence of SNPs on the effect of different treatment strategies based on MRD levels

The MRD in the marrow of pediatric ALL samples with different *NOL1* rs3764909 and *NSUN4* rs10252 alleles after treatment with Chinese Children Cancer Group chemotherapeutics (CCCGs) or South China Children Leukemia Group chemotherapeutics (SCCLGs) was detected. The differences between varieties of alleles were estimated. Unfortunately, we did not identify the association between rs3764909 or rs10252 varieties of alleles and the sensitivity to CCCG treatment or SCCLG treatment in ALL children (**Table 4**).

**Table 4:** The influence of m5C-related gene polymorphisms on sensitivity to different treatment strategies based on MRD levels.

SNP	Variables	Genotype	MRD in marrow (%, 19 days)				MRD in marrow (%, 33 days)				MRD in marrow (%, 12 weeks)			
			Case (%)		p^a^	Adjusted OR ^a^	Case (%)		p^a^	Adjusted OR ^a^	Case (%)		p^a^	Adjusted OR ^a^
			<0.01	≥0.01		(95% CI)	<0.01	≥0.01		(95% CI)	<0.01	≥0.01		(95% CI)
rs3764909	CCCG-	AA	10 (13.70)	109 (34.71)			15 (11.36)	30 (37.04)			7 (58.33)	3 (25.00)		
	ALL-2015	AC/CC	63 (86.30)	205 (65.29)	0.233	0.682 (0.364–1.279)	117 (88.64)	51 (62.96)	0.078	1.514 (0.954–2.403)	5 (41.67)	9 (75.00)	0.737	1.083 (0.679–1.728)
	SCCLG-	AA	3 (60.00)	7 (21.21)			1 (20.00)	3 (23.08)			0 (0.00)	1 (50.00)		
	ALL-2016	AC/CC	2 (40.00)	26 (78.79)	0.869	1.046 (0.615–1.779)	4 (80.00)	10 (76.92)	0.412	1.215 (0.763–1.934)	2 (100.00)	1 (50.00)	0.156	1.370 (0.887–2.116)
rs10252	CCCG-	AA	27 (36.99)	131 (41.99)			45 (34.09)	42 (51.82)			4 (33.33)	6 (50.00)		
	ALL-2015	AG/GG	46 (63.01)	181 (58.01)	0.813	0.927 (0.494–1.739)	87 (65.91)	39 (48.15	0.333	0.802 (0.513–1.254)	8 (66.67)	6 (50.00)	0.823	1.054 (0.565–1.669)
	SCCLG-	AA	2 (40.00)	13 (40.63)			3 (60.00)	6 (46.15)			2 (100.00)	0		
	ALL-2016	AG/GG	3 (60.00)	19 (59.38)	0.939	0.980 (0.574–1.670)	2 (40.00)	7 (53.85)	0.575	1.143 (0.716–1.825)	0	2 (100.00)	0.954	>999.999 (<0.001, >999.999)

CCCG, Chinese Children Cancer Group; SCCLG, South China Children Leukemia Group; MRD, minimal residual disease; SNP, single-nucleotide polymorphism.

a Adjusted for age and gender.

## Discussion

In this case–control study, the possible relationship of m5C methyltransferase coding gene polymorphisms with pediatric ALL risk from a population in southern China was explored. The results discovered that two of the five selected SNPs, *NOL1* rs3764909 G>A and *NSUN4* rs10252 G>A, were associated with increased pediatric ALL, and the other m5C methyltransferase coding genes SNPs were not related to pediatric ALL risk. This is the first study on the association between m5C methyltransferase coding gene polymorphisms and pediatric ALL susceptibility.

In recent years, it has been reported that epigenetic changes, including DNA methylation, RNA methylation, histone modification, and non-coding RNAs, can promote the progression of ALL (18). Several lines of data have introduced m5C modification as an important regulator in post-transcription. In the study of carcinogenesis, m5C-modified genes have been reported to be associated with a variety of cancers, including bladder cancer (19), hepatocellular carcinoma (20), glioblastoma multiforme (21), and leukemia (22). However, limited pieces of evidence have focused on the function of polymorphisms of m5C genes on disease susceptibility. There is only one available study on the epidemiological assessment of SNPs in the m5C modification core gene and cancer. In July of this year, a case–control study was performed and revealed that two SNPs of YBX1 gene, rs10890208 and rs3862218, may predict a reduction by using the Cox regression model to analyze the association between 13 candidate SNPs of the m5C modifier gene and OS of CRC after chemotherapy (17).

Our study identified that *NOL1* rs3764909 and *NSUN4* rs10252 variants could contribute to the increased pediatric ALL risk. *NOL1* and *NSUN4* are important methyltransferases involved in m5C RNA modification (14). Similar to m6A methylation modification, m5C RNA modification can participate in other biological processes including cell growth, proliferation, apoptosis, and differentiation by affecting RNA translation, nuclear export, and stability (23). *NOL1*, one of the m5C methyltransferases, can generate m5C at C72 of tRNA (24, 25). Hong et al. demonstrated that *NOL1* could bind to the T-cell factor binding element of the cyclin D1 gene promoter and enhance transcriptional expression (26). Interestingly, telomerase can also interact with *NOL1* to affect the transcription of the cyclin D1 gene. *NOL1* can promote tumor proliferation by activating cyclins (26). *NOL1*-E2A fusion was regarded as the pathogenesis of acute leukemia in a case report (27). *NOL1* was also reported to promote hepatocellular carcinoma cell proliferation by TGF-β1/hPVT1/NOP2 pathway (28). *NSUN4* is located at chr1 1p33, as the “writer” of m5C, involved in rRNA methylation. It can mediate mitochondrial protein synthesis by regulating the assembly processing and maturation of mt-ribosome (24, 29). It has been shown to be involved in tumor effects. *NSUN4* promotes the malignant progression of hepatocellular carcinoma (30). *NOL1* and *NSUN4* polymorphisms may be involved in tumor risk-related biological functions by affecting m5C modification of coding and non-coding RNAs (24, 31).

In this study, we found that *NOL1* rs3764909 C>A and *NSUN4* rs10252 G>A were associated with increased ALL susceptibility. *NOL1* has been found to be associated with the pathogenesis of leukemia (27), so *NOL1* SNP variants may contribute to the development of ALL. *NSUN4* can affect tumorigenesis by affecting mitochondrial protein synthesis (24). *NSUN4* rs10252 variants may regulate mitochondrial protein synthesis in ALL cells and increase the risk of ALL.

However, there are some limitations to this study. On the one hand, we did not perform independent experimental studies to verify the relationship between selected SNPs and specific risk factors in children with leukemia. On the other hand, for rs3764909 and rs10252, deeply functional verifications are needed to explain the mechanisms of *NOL1* and *NSUN4* in ALL.

In conclusion, *NOL1* rs3764909 and *NSUN4* rs10252 variants are associated with increased ALL tumor susceptibility. The specific mechanisms by which *NOL1* and *NSUN4* polymorphisms are involved in pediatric ALL susceptibility require further study. Genetic variants in m5C modification coding genes were associated with enhanced pediatric ALL susceptibility and suggested that *NOL1* and *NSUN4* gene polymorphisms might be a potential liquid biopsy biomarker for pediatric ALL.

## Data availability statement

The original contributions presented in the study are included in the article/**Supplementary material**. Further inquiries can be directed to the corresponding author.

## Ethics statement

The studies involving human participants were reviewed and approved by Guangzhou Women and Children’s Medical Center. Written informed consent to participate in this study was provided by the participants’ legal guardian/next of kin.

## Author contributions

XPL and HJ contributed to the conception and design of the study. XW and DD wrote the first draft of the manuscript. DD and MC extracted genomic DNA. XW, YY and SL conducted Taqman PCR. AL and XDL collected samples. XZ performed the statistical analysis. XPL revised the manuscript. All authors contributed to the manuscript revision and read and approved the submitted version.
