# Supplementary material for: Genetic variants in m5C modification core genes are associated with the risk of Chinese pediatric acute lymphoblastic leukemia: A five-center case–control study
Source: Front Oncol. 2023 Jan 9;12:1082525. doi: 10.3389/fonc.2022.1082525 (PMC9868168; doi:10.3389/fonc.2022.1082525)
Supplement: Supplementary file 1 [file Table_1.pdf]

Table S1 Information of tested SNPs

SNPs ID	ASSAY ID	Gene	Location	Assay Type	DYE（VIC/FAM）
rs3764909	C_2655176_20	NOL1	Chr12: 6568031	Functionally Tested	A/C
rs7653521	C_29377988_10	NSUN3	Chr3: 94065715	Functionally Tested	C/T
rs10252	C_7567589_10	NSUN4	Chr1: 46364758	Functionally Tested	A/G
rs1880948	C_11996675_20	NSUN5	Chr7: 73308984	Functionally Tested	A/G
rs3740102	C_25641482_10	NSUN6	Chr10: 18651228	Functionally Tested	A/C
